# Phenotypic and Agromorphological Diversity Reveals Detailed Information About Accessions with Productive Potential for Cotton (*Gossypium barbadense* L.) in Northeastern Peru

**DOI:** 10.3390/plants15020314

**Published:** 2026-01-21

**Authors:** Deyvis Córdova-Sinarahua, Susan Linares-Huapaya, Emma Imelda Manco-Céspedes

**Affiliations:** 1 Dirección de Recursos Genéticos y Biotecnología, Estación Experimental Agraria El Porvenir, Instituto Nacional de Innovación Agraria (INIA), Carretera Marginal Sur Fernando Belaúnde Terry KM 13.5, Tarapoto 22400, Peru; linareshs10@gmail.com; 2 Center of Biotechnology and Genetics, Department of Biological Sciences, Universidade Estadual de Santa Cruz, Rodovia Jorge Amado km 16, Ilheus 45662-900, Brazil

**Keywords:** *Gossypium barbadense* L., genotypic coefficient of variation, phenotypic coefficient of variation, heritability, multivariate analysis

## Abstract

Cotton (*Gossypium barbadense* L.) is a crop of great economic importance, as the superior quality of its fiber is highly valued worldwide. The objective of this research was to evaluate the agromorphological diversity of cotton germplasm using both qualitative and quantitative descriptors. A combination of univariate and multivariate statistical analyses was applied to identify promising accessions. The coefficients of variation (GCV and PCV) and high heritability estimates observed for descriptors such as the number of sympodial branches, fiber weight, and number of seeds per capsule confirm genetic control, ensuring the effectiveness of selection in future breeding programs. Correlation analysis revealed a strong positive relationship between lint yield and number of bolls per plant (0.893). Furthermore, principal component analysis indicated that accessions PER1010536, PER1010538, PER1010543, and PER1010547 were associated with high yield and early-maturity traits. Furthermore, multiple correspondence analysis and mixed data factor analysis demonstrated that the observed variability also depends on qualitative traits such as petal spot and bract color, supporting the concept of a complex genetic architecture. These findings provide a solid basis for the development of new cotton cultivars with improved productivity.

## 1. Introduction

Cotton is a major commercial crop of great economic importance, providing the textile industry with the largest source of natural, versatile, and renewable fiber. In addition, the seed is a significant source of vegetable oil and protein, providing essential nutrients for both human and animal diets [1,2,3]. This crop belongs to the genus *Gossypium* of the Malvaceae family and includes both diploid and tetraploid species. Among the latter, two species account for more than 97% of global fiber production: *G. hirsutum* L. (2n = 4x = 52) and *G. barbadense* L. (2n = 4x = 52) [4,5].

*G. barbadense* is a tropical perennial plant native to the Peruvian coast [6], with evidence of its use in textiles dating back thousands of years in pre-Incan cultures. Currently, its fiber is valued for its excellent quality—extra-long, strong, soft, lustrous, and fine—giving producers of specialty cotton a competitive advantage [7,8,9]. Likewise, native cotton, which has been preserved in the Peruvian Amazon through the sociocultural practices of local communities, has enabled the conservation of naturally colored cotton [10,11]. This represents an ecological and low-cost production alternative for the textile industry [12]. Native cotton is cultivated in northern Peru, particularly in the regions of Lambayeque, Piura, and San Martín [13].

The remarkable characteristics of *G. barbadense* are offset by low yield and the challenges posed by climate change. Furthermore, although the study by [3] demonstrated phenotypic variability in *Gossypium* spp., it also highlights the limited number of morphological characterization studies of native cotton, *G. barbadense*. In this context, it is essential to conduct an agromorphological characterization of the accessions in the cotton germplasm collection maintained by the Germplasm Bank of the National Institute of Agrarian Innovation (INIA), to determine their phenotypic and genotypic variability. Thus, 62 descriptors were evaluated to identify the categorical descriptors and their usefulness in distinguishing accessions with higher yield [14], which could be used in future genetic improvement programs.

## 2. Results

### 2.1. Genetic Variability

Analysis of variance (ANOVA) (Table 1) revealed highly significant differences (*p* ≤ 0.01 and *p* ≤ 0.001) among the 34 accessions for all traits except those related to seeds, such as SL and SWi. These differences were also detected throughout the years of evaluation, reflecting the influence of environmental conditions on plant performance. Highly significant differences were observed between accessions in several key traits, such as plant height (PH), number of sympodial and monopodial branches (NSP, NMP), days to fifty percent squaring (DFS), days to fifty percent flowering (DFF), and days to fifty percent pod opening (DBO), capsule length and width (BL, BWid, BLWR), number of seeds per capsule (NSB), seed shape index (SSI), seed weight (SW, SP), and traits related to cotton yield (SCY, SCWP, BW) and fiber weight (FW, GTP, GR). Similarly, the interaction between access and year showed the same pattern of significant differences, while seed dimensional traits (SL and SWi), which showed no significant differences, remained stable throughout the years of evaluation.

The variability of quantitative descriptors was evaluated in the 34 accessions (Table 2). According to this criterion proposed by [15], the variables that showed high PCV values were NSP, Plen, SWi, SL, and SSI. NSP showed high values for both GCV and PCV. Moderate GCV and PCV values were found for LA, DFS, NSB, BW, SL, FW, and GR. In contrast, DTTL, NMP, PH, LL, LW, LI, DFF, CL, DBO, BL, Bwid, BLWR, NLP, DH, and SP corresponded to low GCV and PCV values. In terms of heritability, the categorization indicated by [16] showed that most traits had very high heritability (DFS, DFF, PLen, BL, Bwid, BLWR, NSB, and GR) and moderately high heritability (NSP, NMP, PH, PL, LL, LA, CL, DBO, BW, SW, SP, FW, and GTP). In addition, genetic progress (GA) and genetic progress as a percentage of the mean (GAM) were estimated to complement the heritability estimates. High GAM values were observed for NMP (83.03%), PLen (70.28%), NSB (65.41%), FW (59.21%), DFS (48.84%), BW (45.55%), GR (39.21%), LA (38.28%), and DFF (35.57%). Moderate GAM values were recorded for PH (23.86%), PL (27.74%), BWid (24.43%), BLWR (29.37%), SW (26.06%), SP (21.27%), NSP (20.17%), and GTP (32.50%). In contrast, low GAM values (<10%) were observed for DTTL, LW, LI, NLP, DH, SL, SWi, and SSI.

### 2.2. Association of Agromorphological Descriptors

**Figure 1:**
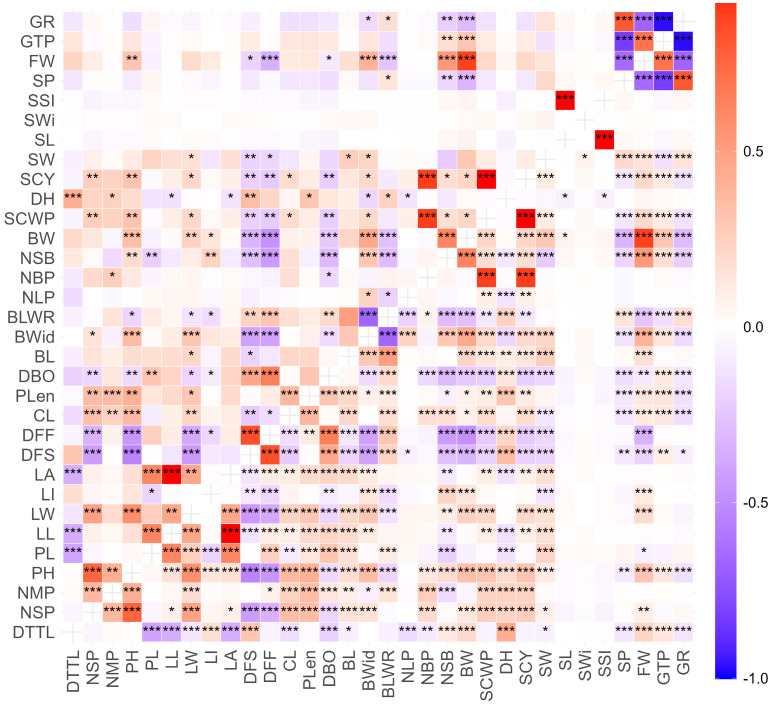
Pearson correlation matrix for 32 agromorphological traits evaluated in 34 cotton accessions from the INIA cotton germplasm collection. Phenotypic correlation coefficient (lower diagonal), genotypic correlation coefficient (upper diagonal). Significant at 5% (*). Significant at 1% (**). Significant at 0.1% (***).

The analysis of phenotypic and genotypic correlation coefficients for agromorphological descriptors revealed highly significant positive and negative correlations (Figure 1). At both the phenotypic and genotypic levels, a strong and highly significant positive correlation was observed between traits related to cotton yield, such as SCY with NBP (0.893), between SCWP and NBP (0.893), and between BW and FW (0.881) and NSB (0.623), as well as between FW and GTP (0.701) and NSB (0.530). Highly significant positive relationships were also observed between leaf descriptors, such as LL with PL (0.624) and LA (0.995), and between LA and PL (0.620); descriptors related to earliness, such as DFF with DFS (0.844) and DBO (0.653); seed descriptors, such as SL with SSI (0.999) and SP with GR (0.807); and between PH and NSP (0.762) and LW (0.574).

On the other hand, highly significant negative correlations were found between GTP and GR (0.968) and SP (0.820), FW and SP (0.638) and GR (0.669), as well as between DFS and NMP (0.537) and between BLWR and LI (0.621).

### 2.3. Multivariate Analysis Using Quantitative and Qualitative Data

**Figure 2:**
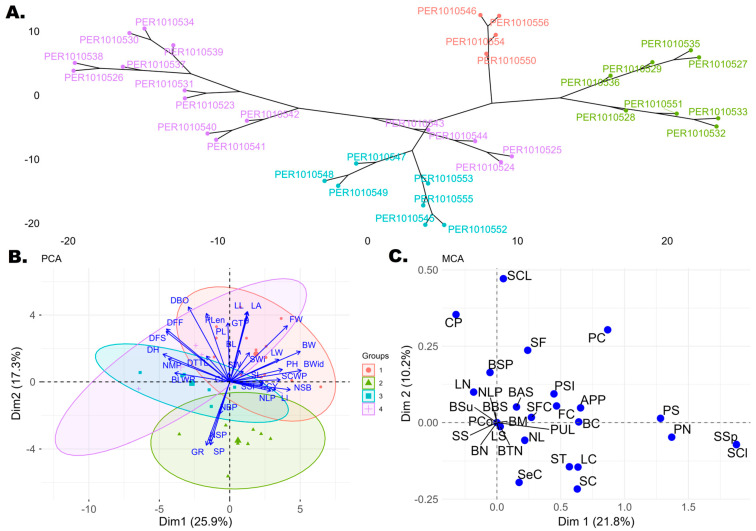
(**A**) Hierarchical dendrogram based on Gower’s distance matrix of the 34 cotton accessions, defining four groups (groups 1–4). In the dendrogram, group 1 is represented in purple, group 2 in green, group 3 in blue, and group 4 in red. (**B**) Principal component analysis (PCA) biplot of Dim 1 versus Dim 2 showing the divergence of quantitative descriptors and the 34 cotton accessions. The same four groups defined in the dendrogram are shown, with Group 1 represented in red, Group 2 in green, Group 3 in blue, and Group 4 in purple. (**C**) Multiple correspondence analysis (MCA) biplot of the 34 cotton accessions based on qualitative descriptors.

Principal component analysis (PCA) of the quantitative traits of the 34 accessions evaluated revealed 31 principal components (PCs), of which five represented a cumulative variability of 71.03%. Dim1 showed a variability of 25.94%, with the largest contributions from BWid (8.75%), BW (8.47%), and DH (7.59%). Dim2 accounted for 17.25% of the variability, mainly represented by DBO (10.26%), LL (9.03%), LA (8.92%), and Plen (8.54%). Dim3 showed a variability of 12.19%, with predominant contributions from SW (13.04%), SP (11.75%), GTP (10.81%), and GR (10.71%). Dim4 explained 9.98% of the variability, represented by NBP (19.12%), CL (15.52%), SCWP (10.65%), and SCY (10.65%). Finally, Dim5 accounted for 6.57% of the variability, with high contributions from LI (17.00%) and BL (14.91%). On the other hand, the hierarchical dendrogram grouped the 34 accessions into four main clusters (Figure 2A), reflecting a considerable degree of divergence among the materials evaluated. These four groups were used as a reference in the PCA biplot, allowing the distribution of the accessions to be visualized in the two dimensions. Consequently, the PCA biplot (Figure 2B) shows four groups (red, green, blue, and purple), illustrating how the 34 accessions relate to the 32 quantitative descriptors. Group 1, represented in red in the PCA biplot (and purple in the dendrogram; Figure 2A) included accessions PER1010526, PER1010538, PER1010530, PER1010534, PER1010537, PER1010539, PER1010531, PER1010523, PER1010540, PER1010541, PER1010542, PER1010543, PER1010544, PER1010525, and PER1010524, which showed high values for the descriptors LL, LA, FW, BW, BWid, SCWP, SCY, and NSB. Group 2 (green) comprised accessions PER1010533, PER1010532, PER1010528, PER1010535, PER1010527, PER1010529, PER1010551, and PER1010536, which showed close relationships between SP, NSP, GR, and NBP. Group 3 (blue) included accessions PER1010553, PER1010549, PER1010548, PER1010547, PER1010552, PER1010545, PER1010555, and PER1010544, closely associated with BLWR, NSP, and DH. Group 4, represented in red in the dendrogram (Figure 2A) and in purple in the PCA biplot (Figure 2B), contained accessions PER1010550, PER1010554, PER1010546, and PER1010556, characterized by higher values of DH, DFF, DFS, BLWR, NMP, and DBO.

In turn, multiple correspondence analysis (MCA) performed on the 30 qualitative descriptors revealed that 32.01% of the variability was explained by the first two dimensions, Dim1 (21.84%) and Dim2 (10.17%). Associations were observed (Figure 2C) between several descriptors: SCl, SSp, and PN, and, to a lesser extent, PS. Accessions PER1010550, PER1010554, PER1010546, and PER1010556 were associated with these descriptors, showing a unique trait profile. Another group of descriptors, SCL and CP, was associated with accessions PER1010533, PER1010551, PER101053, PER101032, PER101035, PER101027, PER101028, and PER101029.

**Figure 3:**
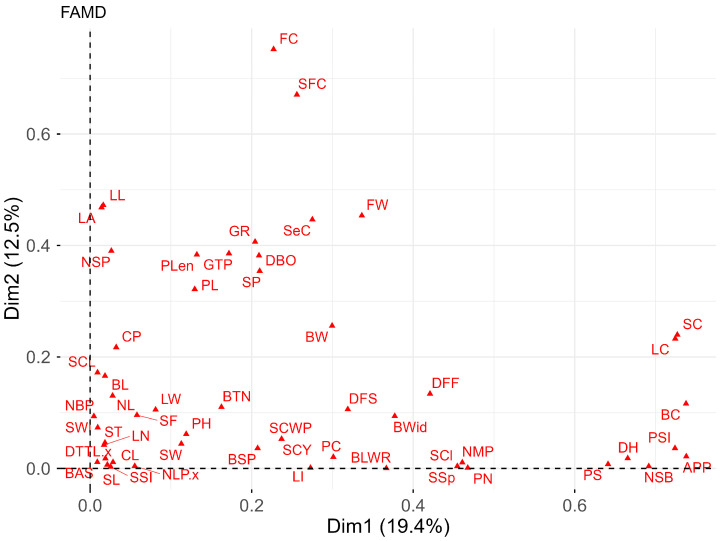
Biplot of mixed data factor analysis (FAMD) for qualitative and quantitative descriptors of the 34 cotton accessions.

Finally, mixed data factor analysis (FAMD) summarizes the relationship between qualitative and quantitative variables. It was observed (Figure 3) that 19.39% of the total variance is explained by dimension 1 and 12.50% by dimension 2. Among the key descriptors for differentiating accessions are quantitative traits (S.1A): FW, NSB, DH, GR, and DBO, and qualitative traits (S.1B): SC, LC, BC, PSI, APP, FC, and SFC.

## 3. Discussion

The germplasm of *G. barbadense* from northeastern Peru showed distinctive variations in its agromorphological traits. Analysis of variance revealed wide variability among accessions, with significant differences in most quantitative traits, confirming the potential of this germplasm for genetic improvement programs. Only seed size traits (SL, SWi, SSI) remained stable, suggesting more conserved genetic control over these characteristics, while the significant interaction between accession and year reflects the influence of the environment on trait expression. In this regard, phenotypic diversity and phenotypic plasticity have been reported to be key factors in selecting outstanding genotypes and improving this crop [17].

Similarly, both the phenotypic coefficient of variation (PCV) and the genotypic coefficient of variation (GCV) allowed us to quantify the variability of traits among germplasm accessions. These estimates suggest that descriptors with PCV values slightly higher than GCV values are less influenced by the environment and, therefore, their variability is mainly genetic [18]. This was observed in days to 50% square (DFS), ginning ratio (GR), number of seeds per capsule (NSB), capsule weight (BW), fiber weight (FW), pedicel length (Plen), and number of sympodial branches (NSP), all of which showed moderate to high PCV and GCV values. Similar results have been reported for BW and NSP [18] and NSB [19]. Similarly, the heritability of the seven descriptors mentioned above ranged from moderately high to very high. Since they are less influenced by the environment, their selection could be relatively straightforward due to the high additive effect on their expression [18,20].

Therefore, they can be improved using simple selection methods, such as direct selection in the first generations [21] or hybridization [22]. Similar patterns have been described for traits related to flowering, pod weight (BW), number of fruiting branches (NSP), number of seeds per pod (NSB) [19], and ginning index [23]. Favorable values for heritability and genetic variability indicate the effectiveness of selection for the genetic improvement of these traits.

Given that several traits showed genetic variability, it is important to understand their interactions with variables related to yield (capsule weight, fiber weight, and raw cotton yield), phenological traits (days to flowering and harvest), and architectural traits, such as the number of branches and leaf area. In this regard, the analysis of genotypic and phenotypic correlation coefficients helped identify significant associations between variables that directly affect cotton yield. Positive correlations were observed between the number of seeds per boll (NSB) and two key components of yield: fiber weight (FW) and boll weight (BW). This indicates that a higher number of seeds leads to an increase in fiber production and total boll weight, suggesting that a higher number of seeds per boll may be related to a reduction in the number of unfertilized ovules per boll [24], i.e., reproductive efficiency that positively impacts cotton yield. Similar results have been obtained between NSB, BW, and fiber yield [25]. Regarding the positive correlation between plant height (PH) and the number of sympodial branches (NSP), as well as leaf width (LW), similar findings have been reported between PH and NSP [23], in addition to their positive effect on cotton yield. In addition, taller plants with larger leaves can achieve greater photosynthetic efficiency, which would have an impact on final yield [26]. Strong correlations were also found, both at the genotypic and phenotypic levels, between seed cotton yield (SCY) and seed cotton weight per plant (SCWP), and with the number of bolls per plant (NBP), as well as between SCWP and NBP. This indicates that a greater number of reproductive structures and a greater mass of lint per plant contribute significantly to increased yield. These findings are consistent with those reported by [26], who observed a very strong association between fiber per boll and cotton weight per boll, indicating that heavier bolls produce more individual fiber, which has a positive impact on total yield. Regarding negative correlations, those observed between the boll-to-leaf ratio (BLWR) and the leaf-to-boll ratio (LI) indicate possible competition in resource allocation. These opposing phenotypic relationships have been described in studies on cotton, such as [27], in relation to source–sink dynamics and resource allocation between vegetative and reproductive structures [28].

In addition to phenotypic and genotypic correlations, multivariate analyses (PCA, MCA, and FAMD) were used to support the interpretation of patterns of variability among accessions by integrating multiple quantitative and qualitative traits. Rather than reiterating individual results, these approaches allowed for the identification of trait syndromes with potential agronomic relevance. PCA indicated that the principal axes were associated with traits related to yield, plant architecture, and phenological development, highlighting the multidimensional nature of cotton variability. Accessions were grouped according to combinations of vegetative vigor, reproductive allocation, and timing of development, reflecting distinct biological strategies. In particular, one group was characterized by greater leaf area and heavier bolls, traits commonly associated with biomass accumulation and yield potential. Comparable associations between capsule traits and yield components have been described for BW, FW, and BWid [29], as well as for SCY and BW [28,30,31,32,33]. The positive relationship between leaf area and yield, previously described by [34,35], suggests that increased photosynthetic capacity may indirectly contribute to productivity. These accessions represent promising material for breeding programs that aim to improve yield through indirect selection using leaf traits [36]. Another group was defined mainly by reproductive efficiency, integrating variables related to sympodial branches, number of capsules, seed percentage, and threshing ratio. Similar relationships between NSP and NBP have been described [37], supporting the role of fruiting structures in determining reproductive yield. These traits are particularly relevant for selection strategies focused on seed production efficiency and pod retention. Variability in phenological behavior was also evident, with accessions differing in maturity patterns and branch architecture. The association of days to harvest with branch type and capsule-related traits supports previous findings indicating that cotton maturity is influenced by plant architecture and fruiting habit [38]. The coexistence of early and late materials within this group may be advantageous for breeding programs targeting production cycles adapted to specific environmental or management conditions [39,40]. Finally, a distinct group was predominantly associated with phenological descriptors related to flowering, capsule formation, capsule opening, and harvest timing. These traits are closely related to crop maturity and the timing of reproductive development. Ref. [41] has described similar contributions of DBO, DFF, and DFS to maturity differentiation. The predominance of late-maturing accessions within this group highlights their potential use in breeding programs aimed at extending the production cycle or adapting cultivars to environments with longer growing seasons.

Complementarily, Multiple Correspondence Analysis (MCA) applied to qualitative variables explained 32.01% of the variability in its first two dimensions. In Dim1 (21.84%), a strong association was observed between descriptors such as seed cleft (SCl), seed shape (SSp), petiole nectaries (PN), and petal spot (PS). In this regard, the study by [15] revealed the high contribution of PS in determining the categorical traits of cotton. The accessions associated with these descriptors were PER1010550, PER1010554, PER1010546, and PER1010556, which showed a morphological profile that could be useful for identifying and characterizing materials with distinctive traits [15]. Another important association was detected between seed coat luster (SCL) and pulvinus color (CP), involving accessions PER1010533, PER1010551, PER1010535, PER1010532, PER1010536, PER1010527, and PER1010528, which overlap with the accessions highlighted in the PCA. This indicates that certain accessions simultaneously concentrate agronomic and qualitative traits of interest.

Finally, mixed data factor analysis (FAMD) integrated quantitative and qualitative variables, explaining 19.39% and 12.50% of the variability in the first two dimensions, respectively. Among the most influential descriptors were, among the quantitative traits, fiber weight, days to 50% capsule opening (DBO), days to harvest (DH), ginning ratio (GR), and number of seeds per capsule (NSB), and among the qualitative traits, stem color (SC), leaf color (LC), bract color (BC), petal spot (PS), petal spot intensity (PSI), presence of anthocyanin in the petiole (APP), fiber color (FC), and lint color (SFC). This integration shows that the variability observed does not depend exclusively on one type of descriptor, but rather on the interaction between productive and morphological traits, as observed in the study by [42], where genetic groups affecting both fiber quality and yield were identified in the same genomic locus. In this sense, accessions such as PER1010536, PER1010538, PER1010543, and PER1010547 emerge as strategic materials for breeding programs focused on yield and precocity, while PER1010550, PER1010554, and PER1010556 stand out for their distinctive morphological attributes.

The Germplasm of *G. barbadense* from northeastern Peru showed distinctive agromorphological variability in all traits evaluated. Analysis of variance revealed wide variation among accessions, with significant differences in most quantitative traits, confirming the potential of this germplasm for cotton breeding programs. In particular, traits showing high genetic variability and heritability, such as boll weight (BW) and number of seeds per boll (NSB), suggest that these characteristics can be effectively improved through direct selection in early generations, increasing fiber productivity and overall yield [23,43]. This study also provides a deeper understanding of how genotype × environment interactions influence the phenotypic expression of key traits such as number of sympodial branches (NSP) and ginning ratio (GR), creating opportunities for the development of varieties better adapted to specific environmental conditions. These morphological traits, which are closely related to increased fiber yield, have important implications for the textile industry, as they provide a genetic basis for producing higher quality and stronger fibers, which are necessary for the manufacture of high-performance textiles [44,45].

## 4. Materials and Methods

### 4.1. Plant Material

**Figure 4:**
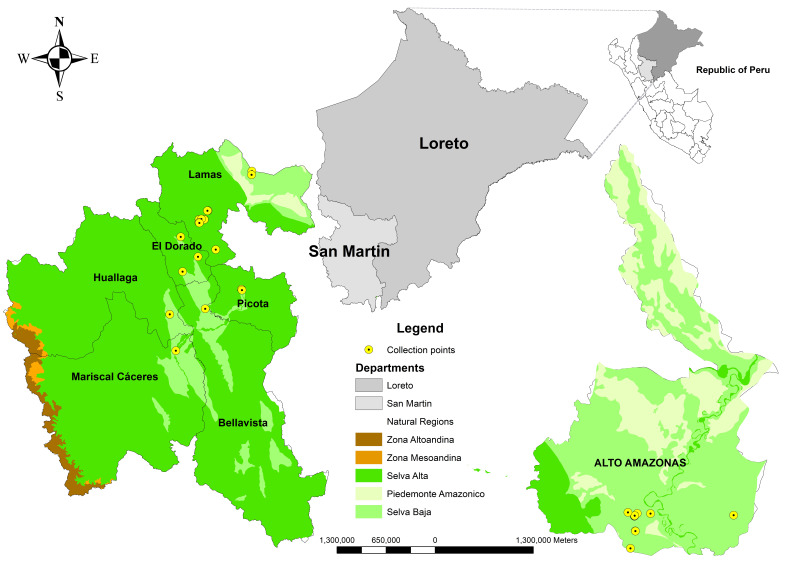
Location of collection sites for the 34 cotton accessions in the departments of San Martín and Loreto.

The plant material was collected in 2019 in the departments of San Martín and Loreto (Figure 4). A total of 34 accessions of *G. barbadense* were collected, mostly from domestic gardens preserved in the surrounding areas. The accessions were sown directly in the field in March, in final experimental plots of 15 m^2^ per accession. Each accession was established in two rows of 11 plants each, giving a total of 22 plants per accession. All accessions were maintained under uniform management conditions, including standardized irrigation, fertilization, and phytosanitary control during the three growing seasons.

### 4.2. Location of the Research

The research was conducted between 2022 and 2024 at the El Porvenir Agricultural Experimental Station of the National Institute of Agricultural Innovation (INIA). It is located in the district of Juan Guerra, province and department of San Martín, Peru, at a latitude of −6°35′26.5″ and a longitude of −76°19′25.47″, at an altitude of 213 m above sea level. Although all accessions received the same agronomic management, environmental conditions varied throughout the three growing seasons, including specific climatic factors for each year.

### 4.3. Experimental Design

The 34 accessions of *G. barbadense* were established in the same experimental plot, with each accession occupying 15 m^2^ distributed in two rows of 11 plants each (22 plants per accession). The distance between rows was 1.5 m and that between plants was 1.0 m.

From each accession, 10 plants were randomly selected and considered as independent experimental units for morpho-agronomic evaluation in each growing season. The experiment was conducted using a completely randomized design (CRD) in each year (2022, 2023, and 2024), considering each accession as a treatment. Since the evaluations were carried out for three consecutive years on the same plot, but under environmental conditions that varied between seasons, the Year factor was included as a fixed effect in the ANOVA. In addition, the Accession × Year interaction was evaluated to assess the phenotypic stability of the traits across seasons.

### 4.4. Variables Evaluated

A total of 32 quantitative traits and 30 qualitative traits were evaluated for the 34 accessions, following the recommendations and descriptors proposed by [46], and the different degrees of color were determined using the Royal Horticultural Society color chart [47]. Of the 62 descriptors evaluated, 4 corresponded to vegetative traits, 14 to the leaf, 14 to the flower, 15 to the capsule, 11 to the seed, and 4 to the fiber (Table 3). All traits were measured or observed at the appropriate stages of development: vegetative and leaf traits were recorded during the vegetative growth phase, floral traits during flowering, capsule and seed traits at fruit maturity, and fiber traits after ginning. Quantitative traits were measured using standard tools (e.g., ruler, caliper, or balance). Qualitative traits were scored visually following the descriptor guidelines, and color traits were determined using the Royal Horticultural Society (RHS) color chart.

### 4.5. Statistical Analysis

Statistical analyses were performed to characterize the accessions. For quantitative descriptors, a two-way analysis of variance (ANOVA) was applied, considering the effects of accessions, years of evaluation, and their interaction, verifying the normality of the residuals using the Shapiro–Wilk test. From the quadratic means of the ANOVA, genetic parameters such as the genotypic coefficient of variation (GCV) and the phenotypic coefficient of variation (PCV) were estimated according to [48], as well as broad-sense heritability (H^2^) [49]. The categorization proposed by [15] was considered for GCV and PCV values, with low (0–10%), moderate (10–20%), and high (more than 20%) values, while for heritability, the categorization indicated by [16] was considered, with low values (below 40%), medium values (40–59%), moderately high values (60–79%), and very high values (≥80%). Similarly, Pearson’s phenotypic correlations and approximate genotypic correlations derived from variance components were calculated and represented using heat maps with significance levels. To explore the multivariate structure of the data according to the nature of the variables, complementary ordering methods were applied. Principal component analysis (PCA) was performed on the quantitative variables to identify the main gradients of variation and evaluate the contribution and correlation structure of the continuous descriptors.

Multiple correspondence analysis (MCA) was applied to the qualitative variables to explore associations between categories and visualize their relationships in a reduced-dimension space, and a mixed hierarchical cluster analysis based on Gower’s distance [50] and the Ward D2 method was represented with phylogenetic dendrograms. In addition, a mixed-data factor analysis (FAMD) was applied as an integrative approach to jointly analyze quantitative and qualitative variables, providing an overview of the multivariate relationships between accessions. Although FAMD incorporates all the variables analyzed separately using PCA and MCA, the latter methods were retained to obtain specific interpretations of the type of variables that are not fully captured by the mixed analysis alone. All analyses were performed in the R v.4.5.1 programming environment [51], using the ExpDes.pt, FactoMineR, factoextra, ggplot2, cluster, corrplot, tidyverse, and openxlsx packages within the RStudio Version 4.5.2 interface.

## 5. Conclusions

Cotton germplasm from northeastern Peru showed wide variability with highly heritable traits under genetic control, especially capsule weight, fiber weight, number of seeds, and days to flowering, making them reliable descriptors for direct selection. Multivariate analyses identified promising accessions such as PER1010536, PER1010538, PER1010543, and PER1010547 (notable for their yield and earliness), and PER1010550, PER1010554, and PER1010556 (with vegetative vigor and fiber attributes). Similarly, materials such as PER1010550, PER1010554, and PER1010556 stood out for their unique morphological traits, relevant for diversity and conservation. These results confirm the value of these accessions as a basis for cotton breeding programs.

## Figures and Tables

**Table 1:** Analysis of variance (ANOVA) for the 34 cotton accessions evaluated with the 32 quantitative characteristics.

Quadratic Averages				
Descriptor	Accession (gl = 33)	Years (gl = 2)	Accession x Year (gl = 66)	Error Term
DTTL	6.622 ***	930.294 ***	5.446 ***	0.000
NSP	37.938 ***	679.809 ***	8.531 ***	2.967
NMP	59.214	7810.049	15.008	5.744
PH	5873.029 ***	712,261.572 ***	1181.599 ***	228.842
PL	119.536 ***	1219.576 ***	37.052 ***	3.349
LL	50.912 ***	576.530 ***	16.870 ***	2.225
LW	61.707 ***	2551.228 ***	47.165 ***	2.969
LI	0.173 ***	1.266 ***	0.157 ***	0.043
LA	156,816.931 ***	1,700,970.524 ***	49,434.748 ***	6682.961
DFS	2390.897 ***	18,696.863 ***	189.489 ***	0.000
DFF	2574.652 ***	12,419.510 ***	212.540 ***	0.000
CL	4.465 ***	45.822 ***	1.009 ***	0.145
PLen	14.396 ***	175.724 ***	2.389 ***	0.564
DBO	1619.231 ***	6475.392 ***	563.776 ***	0.000
BL	2.055 ***	5.474 ***	0.227 ***	0.085
BWid	1.084 ***	2.396 ***	0.076 ***	0.027
BLWR	0.644 ***	0.067 **	0.033 ***	0.011
NLP	0.056 ***	0.365 ***	0.045 **	0.032
NBP	3112.506 ***	6626.589 ***	2313.594 ***	450.816
NSB	559.607 ***	1253.208 ***	40.278 ***	8.361
BW	15.678 ***	103.319 ***	3.146 ***	0.331
SCWP	57,564.431 ***	484,863.558 ***	44,838.378 ***	9550.431
DH	426.679 ***	5763.824 ***	235.743 ***	0.000
SCY	2,558,677.278 ***	21,551,622.666 ***	1,993,016.971 ***	424,506.023
SW	54.628 ***	15.236 ***	21.343 ***	0.004
SL	1035.989 n.s	744.595 n.s	988.690 n.s	993.642
SWi	325.023 n.s	220.677 n.s	328.050 n.s	319.288
SSI	0.394 ***	2.036 ***	0.260 ***	0.050
SP	506.471 ***	296.153 ***	101.373 ***	11.758
FW	4.599 ***	26.566 ***	1.186 ***	0.077
GTP	469.735 ***	422.925 ***	100.124 ***	9.648
GR	2.857 ***	1.865 ***	0.436 ***	0.049

Abbreviations: DTTL: Days to third true leaf; NSP: Number of sympodia per plant; NMP: Number of monopods per plant; PH: Plant height; PL: Petiole length; LL: Leaf length; LW: Leaf width; LI: Leaf index; LA: Leaf area; DFS: Days to fifty percent squaring; DFF: Days to fifty percent flowering; CL: Corolla length; PLen: Pedicel length; DBO: Days to fifty percent capsule opening; BL: Capsule length; BWid: Capsule width; BLWR: Capsule length/width; NLP: Number of locules per capsule; NBP: Number of capsules per plant; NSB: Number of seeds per capsule; BW: Capsule weight; SCWP: Seed cotton weight per plant; DH: Days to harvest; SCY: Weight of raw cotton per plant; SW: Weight of 100 seeds; SL: Seed length; SWi: Seed width; SSI: Seed shape index; SP: Seed percentage; FW: Fiber weight; GTP: Ginning percentage; GR: Ginning rate. ** Significant at 1%. *** Significant at 0.1%. n.s not significant.

**Table 2:** Descriptive parameters and genetic variation of 32 quantitative traits evaluated in 34 cotton accessions.

Variables	Mean	σ^2^g	σ^2^axb	σ^2^e	σ^2^p	H^2^%	GCV%	PCV%	GA	GAM
DTTL	17.26	0.04	0.54	0.00	0.22	17.77	1.15	2.72	0.33	1.93
NSP	4.20	0.98	0.56	2.97	1.26	77.51	23.58	26.78	4.20	20.17
NMP	20.80	1.47	0.93	5.74	1.97	74.65	5.83	6.75	3.49	83.03
PH	187.40	156.38	95.28	228.84	195.77	79.88	6.67	7.47	44.71	23.86
PL	19.86	2.75	3.37	3.35	3.98	69.00	8.35	10.05	5.51	27.74
LL	21.61	1.13	1.46	2.22	1.70	66.86	4.93	6.03	3.48	16.12
LW	22.50	0.48	4.42	2.97	2.06	23.57	3.09	6.37	1.35	6.01
LI	1.73	0.00	0.01	0.04	0.01	9.24	1.33	4.38	0.03	1.62
LA	517.33	3579.41	4275.18	6682.96	5227.23	68.48	11.56	13.98	198.03	38.28
DFS	67.31	73.38	18.95	0.00	79.70	92.07	12.73	13.26	32.88	48.84
DFF	95.57	78.74	21.25	0.00	85.82	91.74	9.28	9.69	34.00	35.57
CL	7.00	0.12	0.09	0.15	0.15	77.41	4.85	5.51	1.19	17.05
PLen	3.29	0.40	0.18	0.56	0.48	83.41	19.24	21.07	2.31	70.28
DBO	147.19	35.18	56.38	0.00	53.97	65.18	4.03	4.99	19.16	13.01
BL	5.44	0.06	0.01	0.08	0.07	88.97	4.53	4.81	0.93	17.11
BWid	2.89	0.03	0.00	0.03	0.04	92.97	6.34	6.57	0.71	24.43
BLWR	1.89	0.02	0.00	0.01	0.02	94.87	7.54	7.74	0.56	29.37
NLP	3.04	0.00	0.00	0.03	0.00	21.16	0.66	1.43	0.04	1.21
NBP	59.16	26.63	186.28	450.82	103.75	25.67	8.72	17.22	10.46	17.68
NSB	24.51	17.31	3.19	8.36	18.65	92.80	16.98	17.62	16.03	65.41
BW	5.07	0.42	0.28	0.33	0.52	79.93	12.74	14.25	2.31	45.55
SCWP	255.14	424.20	3528.79	9550.43	1918.81	22.11	8.07	17.17	38.74	15.18
DH	170.59	6.36	23.57	0.00	14.22	44.75	1.48	2.21	6.75	3.96
SCY	1700.99	18,855.34	156,851.09	424,506.02	85,289.24	22.11	8.07	17.17	258.25	15.18
SW	12.62	1.11	2.13	0.00	1.82	60.93	8.35	10.69	3.29	26.06
SL	11.32	1.58	0.00	993.64	34.70	4.54	11.10	52.06	1.07	9.48
SWi	5.75	0.00	0.88	319.29	10.94	0.00	0.00	57.50	0.00	0.00
SSI	2.19	0.01	0.01	32.48	1.10	1.34	5.53	47.83	0.06	2.56
SP	61.80	13.50	8.96	11.76	16.88	79.98	5.95	6.65	13.15	21.27
FW	1.96	0.11	0.11	0.08	0.15	74.21	17.18	19.95	1.16	59.21
GTP	38.32	12.32	9.05	9.65	15.66	78.68	9.16	10.33	12.45	32.50
GR	2.67	0.08	0.04	0.05	0.10	84.75	10.65	11.57	1.05	39.21

Abbreviations: σ^2^g: genotypic variance, σ^2^axb: interaction variance, σ^2^e: error variance, σ^2^p: environmental variance, H: broad-sense heritability, GCV: genotypic coefficient of variation, PCV: phenotypic coefficient of variation, GA: genetic advance, GAM: genetic advance as a percentage of the mean.

**Table 3:** Qualitative and quantitative descriptors used in the characterization of tropical cotton accessions.

Descriptor	Acronym	Unit of Measurement	Characteristic
Days until the third true leaf	DTTL	days	QN
Number of sympodiums per plant	NSP	cm	QN
Number of monopodiums per plant	NMP	#	QN
Plant height	PH	#	QN
Stem color	SC		QL
Leaf shape	LS		QL
Pubescence on leaf underside	PUL		QL
Number of lobes	NL		QL
Petiole length	PL	cm	QN
Leaf length	LL	cm	QN
Leaf width	LW	cm	QN
Leaf index	LI	cm	QN
Leaf color	LC		QL
Pullus color	CP		QL
Leaf nectaries	LN		QL
Petiole nectaries	PN		QL
Anthocyanin pigmentation in the petiole	APP		QL
Leaf area	LA	cm^2^	QN
Days to 50% squaring	DFS	days	QN
Days to 50% flowering	DFF	days	QN
Corolla length	CL	cm	QN
Pedicel length	PLen	cm	QN
Number of bract teeth	BTN		QL
Cotton-like bract margin	BM		QL
Bract color	BC		QL
Petal color	PC		QL
Petal spot	PS		QL
Petal spot intensity	PSI		QL
Bract nectaries	BN		QL
Stigma	ST		QL
Stigma shape	SS		QL
Pollen color	PCo		QL
Days until 50% of capsules open	DBO		QN
Capsule shape	BSP		QL
Capsule apex shape	BAS		QL
Capsule apex shape	BBS		QL
Capsule surface area	BSu		QL
Capsule length	BL	cm	QN
Capsule width	BWid	cm	QN
Capsule L/W ratio	BLWR	cm	QN
Number of locules per capsule	NLP	#	QN
Number of capsules per plant	NBP	#	QN
Number of seeds per capsule	NSB	#	QN
Weight of capsules	BW	g	QN
Weight of raw cotton per plant	SCWP	g	QN
Days to harvest	DH	cm	QN
Raw cotton yield	SCY	kg/ha	QN
Seed lint	SF		QL
Color of seed lint	SFC		QL
Color of the seed	SeC		QL
Glossiness of the seed coat	SCL		QL
Seed shape	SSp		QL
Seed cleft	SCl		QL
Weight of 100 seeds	SW	g	QN
Seed length	SL	cm	QN
Seed width	SWi	cm	QN
Seed shape index	SSI	cm	QN
Percentage of seeds	SP	%	QN
Fiber color	FC		QL
Fiber weight	FW	g	QN
Ginning yield percentage	GTP	%	QN
Ginning index	GR	#	QN

QL, qualitative trait; QN, quantitative trait; # indicates the quantity expressed as several units.

## Data Availability

The original contributions presented in this study are included in the article/Supplementary Materials. Further inquiries can be directed to the corresponding author.

## Supplementary Materials

The following supplementary information can be downloaded at: https://www.mdpi.com/article/10.3390/plants15020314/s1. Figure S1: Factor Analysis of Mixed Data (FAMD) for quantitative (**A**) and qualitative (**B**) descriptors, highlighting the contribution of each descriptor to the principal dimensions.
