# Supplementary material for: Phenotypic and Agromorphological Diversity Reveals Detailed Information About Accessions with Productive Potential for Cotton (Gossypium barbadense L.) in Northeastern Peru
Source: Plants (Basel). 2026 Jan 21;15(2):314. doi: 10.3390/plants15020314 (PMC12844782; doi:10.3390/plants15020314)
Supplement: Supplementary file 1 [file plants-15-00314-s001.zip › plants-4018691-supplementary.pdf]

Supplementary Material

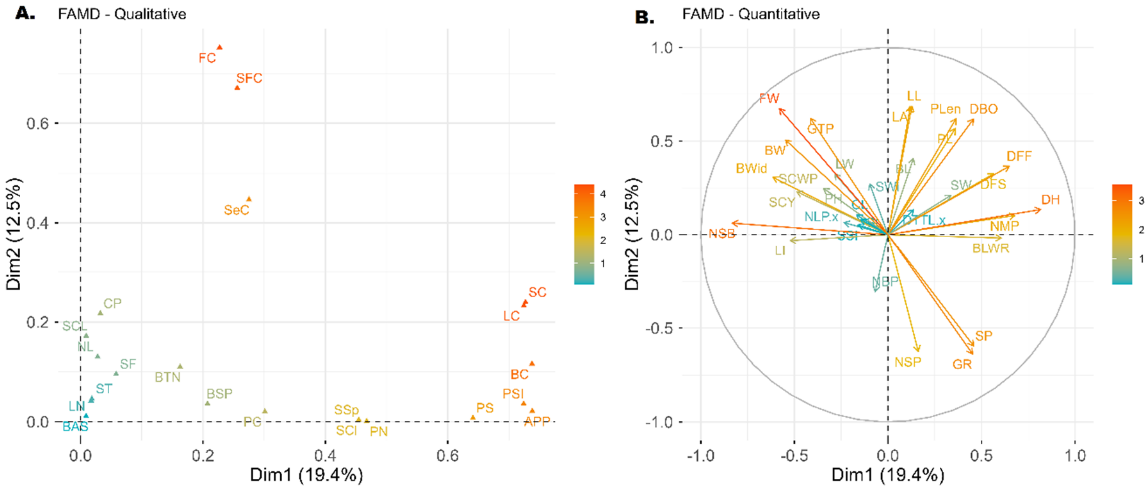

Figure S1: Factor Analysis of Mixed Data (FAMD) for quantitative (A) and qualitative (B) descriptors, highlighting the contribution of each descriptor to the principal dimensions.
